# Prokaryotic Genome Expansion Is Facilitated by Phages and Plasmids but Impaired by CRISPR

**DOI:** 10.3389/fmicb.2019.02254

**Published:** 2019-10-16

**Authors:** Na L. Gao, Jingchao Chen, Teng Wang, Martin J. Lercher, Wei-Hua Chen

**Affiliations:** ^1^Key Laboratory of Molecular Biophysics of the Ministry of Education, Hubei Key Laboratory of Bioinformatics and Molecular-Imaging, Department of Bioinformatics and Systems Biology, College of Life Science and Technology, Huazhong University of Science and Technology, Wuhan, China; ^2^Institute for Computer Science and Department of Biology, Heinrich Heine University, Duesseldorf, Germany; ^3^College of Life Science, Henan Normal University, Xinxiang, China; ^4^Huazhong University of Science and Technology Ezhou Industrial Technology Research Institute, Ezhou, China

**Keywords:** prokaryotic genome expansion, viruses, plasmids, CRISPR, horizontal gene transfer

## Abstract

Viruses and plasmids can introduce novel DNA into bacterial cells, thereby creating an opportunity for genome expansion; conversely, CRISPR, the prokaryotic adaptive immune system, which targets and eliminates foreign DNAs, may impair genome expansions. Recent studies presented conflicting results over the impact of CRISPR on genome expansion. In this study, we constructed a comprehensive dataset of prokaryotic genomes and identified their associations with viruses and plasmids. We found that genomes associated with viruses and/or plasmids were significantly larger than those without, indicating that both viruses and plasmids contribute to genome expansion. Genomes were increasingly larger with increasing numbers of associated viruses or plasmids. Conversely, genomes with CRISPR systems were significantly smaller than those without, indicating that CRISPR has a negative impact on genome size. These results confirmed that on evolutionary timescales, viruses and plasmids facilitate genome expansion, while CRISPR impairs such a process in prokaryotes. Furthermore, our results also revealed that CRISPR systems show a preference for targeting viruses over plasmids.

## Introduction

Gene duplication and/or horizontal gene transfer (HGT) play important roles in functional innovation and species adaptation, and are the main sources of genome expansions (Isambert and Stein, 2009; Schonknecht et al., 2013; Nyvltova et al., 2015; Smith et al., 2016; Tsai et al., 2018). In prokaryotes, it has been shown that the importance of HGT for genome expansions can even outweigh that of gene duplication (Pal et al., 2005; Treangen and Rocha, 2011).

Mobile DNA elements such as viruses and plasmids can introduce novel DNAs into the host genomes (Yamaguchi et al., 2001; Jensen and Lyon, 2009; Lindsay, 2010; Malachowa and Deleo, 2010). They often have a very narrow range of hosts; but under certain conditions, such as antibiotic stress, viruses and plasmids can expand their host ranges (Modi et al., 2013). Therefore, viruses and plasmids are important sources of HGT and of prokaryotic innovations, and consequently drive bacterial evolution and adaptation (Koonin and Wolf, 2008; Nogueira et al., 2009; Argov et al., 2017).

Viruses and plasmids are widely distributed in prokaryotes. Unlike plasmids, viruses are parasites that often lead to lysis of their hosts (Deresinski, 2009; Wernicki et al., 2017). Over the course of prokaryotic evolution, bacteria and archaea developed various defense systems against viruses, plasmids, and other invading genetic elements (Luk et al., 2014). CRISPR (clustered regularly interspaced short palindromic repeats), the adaptive immune system of prokaryotes, is a recently recognized player in the ongoing arms race between prokaryotic viruses and hosts, and plays an important role in the dynamic process by which the genomes of prokaryotes and mobile elements coevolve. CRISPR systems are widespread in prokaryotes, exists in about 40% of bacteria and 90% of archaea (Godde and Bickerton, 2006; Makarova et al., 2011; Seed et al., 2013; Huang et al., 2016), or ∼10% of bacteria as revealed by a recent study (Burstein et al., 2016). CRISPR systems can also target plasmids (Marraffini and Sontheimer, 2008), although plasmids are not necessarily detrimental to their host’s fitness but instead often carry a diverse range of antimicrobial and biocide resistance genes that may help their hosts to survive under certain conditions (Mccarthy and Lindsay, 2012; Shabbir et al., 2016).

Based on the above observations, it is reasonable to speculate that over the course of evolution, viruses and plasmids may contribute to the expansion of prokaryotic genomes, while CRISPR systems may impair such a process. These speculations are consistent with recent observations that CRISPR limits HGT by targeting foreign DNAs (Marraffini and Sontheimer, 2008; Bikard et al., 2012). However, controversial observations have also been reported recently. For example, Gophna et al. (2015) did not observe the expected negative correlation between CRISPR activity in microbes with three independent measures of recent HGT, leading them to conclude that the inhibitory effect of CRISPR against HGT is undetectable. Furthermore, a recent study revealed that CRISPR-mediated phage resistance can even enhance HGT by increasing the resistance of transductants against subsequent phage infections (Watson et al., 2018). These observations appear surprising, as the restricted acquisition of foreign genetic material is believed to be one of the sources of the maintenance fitness cost of CRISPR systems and may be one of the reasons for the patchy distribution of CRISPR among bacteria (Frost et al., 2005; Baltrus, 2013). Thus, it is currently unclear what long-term effects CRISPR, viruses, and plasmids have on genome expansion.

In this study, we first collected a comprehensive dataset of prokaryotes and their associations with viruses, plasmids, and CRISPR systems. We then evaluated the contributions of viruses, plasmids, and CRISPR to genome size. After controlling for genome GC (guanine+cytosine) content, which is known to correlate significantly with genome size (Chen et al., 2016a,b), small genome size typically exhibits low GC content, and this bias in base composition has been explained as consequences of genome recoding and selection on efficient resource usage. However, one example is thermophiles, preferentially grow in high heat conditions, which have much more G/C pairs in the coding regions to enhance the stability of mRNA secondary structure (Basak et al., 2010), and decreased genome size to limit their cost of living (Sabath et al., 2013). The evolutionary forces constraining genome size and GC-content have been attributed to a variety of factors, such as environmental energetic constraints. We found that both viruses and plasmids are associated with larger genomes, while the presence of a CRISPR system is associated with small genome size. Genome sizes increase with increasing numbers of associated viruses and plasmids. Our results clearly indicate that in the long run, viruses and plasmids facilitate genome expansions, while CRISPR impairs such a process in prokaryotes. Furthermore, our results also reveal a striking preference of CRISPR systems for targeting viruses rather than plasmids, consistent with the typical consequences of phage and plasmid infections to the hosts and the roles of CRISPR as a defense system.

## Materials and Methods

### Data

We obtained data from three sources. Microbe-phage interaction data was collected from the MVP database, which we described in a previous publication (Gao et al., 2018). MVP is one of the latest and largest databases about microbe-phage interactions, which supplied 26,572 interactions between 9,245 prokaryotes and 18,608 viral clusters based on 30,321 evidence entries (Gao et al., 2018).

The basic genome information from complete archaeal and bacterial genomes, including the number of associated plasmids, was downloaded from the NCBI Genome database^1^ (N.R. Coordinators, 2018). In order to remove redundancy and avoid incomplete annotation, we only used the complete closed genomes in this study, which represented only a small part of all genome drafts (mostly incomplete) available from NCBI. We obtained in total 14,575 complete prokaryotic genomes (340 archaeal and 14,286 bacterial genomes) and belonging to 7,151 species. We selected a represented genome for each of species with the highest GC-contents among the strains. Among which, 2,287 prokaryotes were identified associating with plasmids.

The CRISPRs data was obtained from the CRISPRCasDb database^2^ (Grissa et al., 2007; Couvin et al., 2018) including 340 archaeal and 16,650 bacterial strains. 2,927 complete prokaryotic genomes (231 archaeal and 2,696 bacterial genomes) were associated with CRISPR systems, while 66 encode CRISPR exclusively on plasmids. The 66 genomes which only contained plasmid-encoded CRISPR systems were removed from all analyses.

In total, 7,085 prokaryotes were found in both of the first two datasets; among these, 2,221 contained plasmids, 2,682 contained viruses, and 2,861 contained CRISPRs on their chromosomes. Detailed information on the dataset can be found in Supplementary Table 2.

### Statistical Analysis

All data were analyzed using R v3.4 (R Core Team, 2017). All pair-wise comparisons between two groups of numeric data (genome sizes or genomic GC-contents) were performed by Wilcoxon rank-sum tests. Linear model (LM) analysis was performed with the R function glm. Relative importance analysis was performed with the calc.relimp function available from the R package “relatimpo” (Groemping, 2006).

## Results

### Prokaryotic Genomes and Their Associations With Viruses, Plasmids and CRISPRs

To systematically investigate the impacts of viruses, plasmids, and CRISPRs on genome expansion, we constructed a list of 7,085 completely sequenced prokaryotic genomes and obtained their associations with viruses, plasmids, and CRISPRs; for details please consult the section “Materials and Methods” and Supplementary Table 2.

**FIGURE 1:**
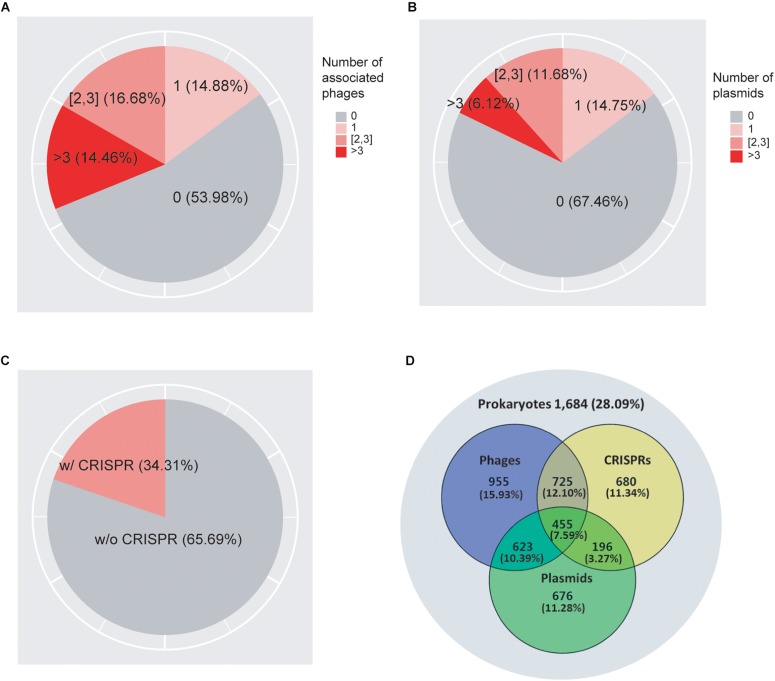
A total of 7,085 prokaryotic genomes and their associations with viruses **(A)**, plasmids **(B)**, and CRISPRs **(C)**. The Venn diagram **(D)** shows the overlap of their distributions in prokaryotes. 1,962 genomes (27.69%) were not found to be associated with viruses, plasmids, or CRISPRs; 439 (6.2%) genomes were associated with all three elements.

As shown in Figure 1A, we found that 62.15% of prokaryotes had no known associations with infecting viruses. 12.24, 13.62, and 12% of prokaryotes were associated with one, two to three, and more than three viruses, respectively. In addition, we found that 68.02% of prokaryotes did not associate with plasmids, while 15.13, 11.12, and 5.73% of the genomes associated with one, two to three, and more than three plasmids, respectively (Figure 1B). Previous studies suggested that the genomic GC-contents as well as nucleotide frequencies of phages and plasmids often closely resembles that of their hosts (Nakashima et al., 2015; Ahlgren et al., 2017; Ren et al., 2017); consistent with these previous observations, we obtained correlation coefficient values of 0.969 and 0.968 between the GC-contents of the host genomes and their associated viruses and plasmids, respectively (Supplementary Figures 1A,B), confirming the high quality of our association data. We found that in total 40.44% of genomes collected in this study contained either viruses or plasmids but not both, while 14.39% of genomes contained both viruses and plasmids (Figure 1D).

As shown in Figure 1C, we found CRISPR systems in 40.38% of the prokaryotic genomes; this percentage is within the range of previously reported numbers (Godde and Bickerton, 2006; Makarova et al., 2011; Seed et al., 2013; Burstein et al., 2016; Huang et al., 2016). We found that CRISPRs were significantly enriched in virus-associated compared to non-virus-associated genomes (odds ratio OR = 1.18, *P* = 1.07 × 10^–3^ from Fisher’s exact test) but not in plasmid-associated compared to non-plasmid-associated genomes (OR = 1.04, *P* = 0.43). In addition, we found that CRISPRs were enriched in virus-associated compared to plasmid-associated genomes, although the significance was only marginal (OR = 1.15, *P* = 0.08, excluding genomes containing both viruses and plasmids), suggesting a strong target preferences of CRISPRs toward viruses (Table 1).

**TABLE 1:** Estimated enrichment of CRISPR in virus-associated and plasmid-associated genomes compared to other genomes, and enrichment of CRISPR in virus-associated compared to plasmid-associated genomes.

**Comparison**	**Odds ratio^b^**	***P-*value^c^**
Virus-associated vs. others	1.18	1.07 × 10^–3^
Plasmid-associated vs. others	1.04	0.43
Virus- vs. plasmid-associated	1.08	0.21
Virus-associated vs. others^a^	1.17	7.48 × 10^–3^
Plasmid-associated vs. others^a^	0.97	0.67
Virus- vs. Plasmid-associated^a^	1.15	0.08

*^*a*^excluding genomes contained both viruses and plasmids. ^*b*^odds ratio OR > 1 indicates enrichment of CRISPR in the first group, while OR < 1 indicates depletion. ^*c*^*P-*values indicate whether CRISPR is significantly enriched or depleted in the first group as compared with the second according to Fisher’ exact test.*

### Viruses and Plasmids Are Associated With Larger Genomes, While CRISPR Is Associated With Smaller Ones

We next investigated which factors contribute significantly to genome size. Previous results have shown a strong correlation between genomic GC content and genome size (Chen et al., 2016a); GC content may even play a causal role in shaping genome size (Chen et al., 2016b). Applying a (LM, see section “Materials and Methods” for details), we found that GC content was indeed the strongest predictor of genome size (Table 2). The LM analysis also revealed that the presence/absence of viruses, plasmids, and CRISPR all significantly influenced genome size; the presences of viruses and of plasmids were associated with increased genome sizes, while CRISPR was associated with decreased genome sizes (Table 2). We estimated that the relative importance of these factors for genome size were 89% for GC-content, 6.11% for virus presence, 3.22% for plasmid presence, and 0.04% for CRISPR presence. This revealed that GC-content was indeed the most significant predictor of genome size; the presence of plasmids and viruses also had a significant influence on genome size; as compared with other factors, the presence/absence of CRISPR had relative small influence on genome size. Interestingly, we found that the presence of both viruses and plasmids in the same genome was associated with a smaller genome size than expected (i.e., the interaction term viruses^∗^plasmids was negative, Table 2). We hypothesized that there are fitness costs inherent to expanding or limiting the genome size, when a given prokaryote is in a highly diverse and competitive environments. In addition to the CRISPR systems, there are other known and novel anti-phage defense systems in the microbial pan-genome (Doron et al., 2018). Unless stated otherwise, we thus limit our further analyses to prokaryotes that contained either viruses or plasmids but not both. Note that our conclusions on the influence of viruses, plasmids, and CRISPR systems on genome size remain unchanged if we perform separate analyses on genomes containing no viruses and on genomes containing no plasmids (Table 2).

**TABLE 2:** Relative importance of various factors for genome size in a linear model (LM).

**Dataset**	**Factor**	**Coefficient**	***P-*value**	**Relative importance**
All	GC%	0.086	<2 × 10^–16^	91.84%
	Plasmid	0.714	<2 × 10^–16^	5.91%
	Virus	0.454	<2 × 10^–16^	2.22%
	CRISPR	−0.043	0.248	0.03%
	Virus^∗^plasmid	−0.130	0.104	–
No plasmids	GC%	0.087	<2 × 10^–16^	96.62%
	Virus	0.454	<2 × 10^–16^	3.18%
	CRISPR	−0.108	0.017	0.20%
No viruses	GC%	0.087	<2 × 10^–16^	93.16%
	Plasmid	0.713	<2 × 10^–16^	6.77%
	CRISPR	0.066	0.168	0.07%

*The equation of “All” dataset used in the LM is size ∼ GC% + plasmid + virus + CRISPR + virus^∗^plasmid. Here, size represents the genome size; GC% represents the genomic GC-content of the host genome; plasmid, virus, and CRISPR represent whether the host genomes are associated with plasmids, viruses, and CRISPR, respectively. The “Coefficient” column contains estimated regression coefficients calculated by ordinary least squares. Relative importance was calculated using the “relaimpo” package (Groemping, 2006); the equation of “No plasmids” dataset is size ∼ GC% + virus + CRISPR; and the equation of “No viruses” dataset is size ∼ GC% + plasmid + CRISPR.*

### Increasing Numbers of Viruses and Plasmids Are Associated With Increased Genome Sizes

**FIGURE 2:**
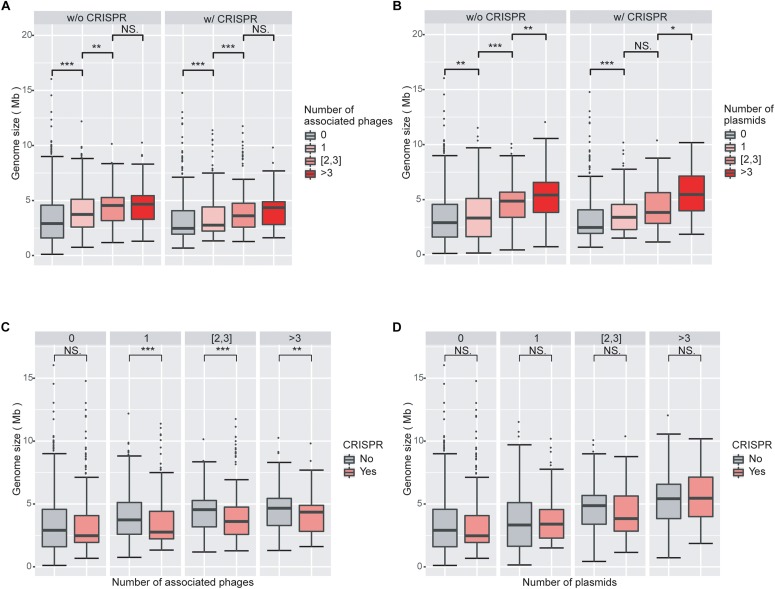
Increasing numbers of viruses and plasmids are associated with increased genome sizes, while virus-associated genomes with CRISPR systems are smaller than those without CRISPR systems. **(A)** Boxplot of genomes size as a function of the number of associated viruses. Genome sizes are larger with increasing numbers of associated viruses, when genomes without CRISPR systems. **(B)** Boxplot of genomes size as a function of the number of associated plasmids. The impact of plasmids on genome size is similar to that of viruses. **(C)** Boxplot of genome size as a function of the presence/absence of CRISPRs in genomes associated with viruses. Virus-associated genomes with CRISPR systems are significantly smaller in size than those without CRISPR, regardless of the number of viruses they are associated with. **(D)** Boxplots of genome sizes in genomes associated with plasmids as a function of the presence/absence of CRISPRs. CRISPRs have no significant impact on genome sizes in genomes associated with plasmids. Wilcoxon rank sum tests were used to compare between groups. Level of significance: ^∗∗∗^*P* < 0.001; ^∗∗^*P* < 0.01; ^∗^*P* < 0.05; NS. *P* ≥ 0.05.

We next investigated the impact of the numbers of viruses and plasmids on genome size. Viruses and plasmids often have very narrow host ranges (Suzuki et al., 2014; Gao et al., 2018); the number of known associations with viruses may indicate the ability of the prokaryotic host to acquire external novel DNA. Consistent with our expectation, we found that genomes associated with more viruses had larger overall genomes (Figure 2A; Supplementary Figure 2A). We observed similar results with plasmids (Figure 2B; Supplementary Figure 2B).

Consistent with the results from the LM analysis, we found that virus-associated genomes are statistically significantly smaller when they encode a CRISPR system compared to when they do not (Figure 2C). However, we did not find a corresponding trend in plasmid-associated genomes (Figure 2D). These results are consistent with the different fitness consequences of virus and plasmid invasions to the prokaryotic hosts. Both viruses and plasmids can bring exogenous DNA to prokaryotes and decrease the fitness of their hosts, for example by increasing the burden on the host’s transcription and translation apparatus. However, viruses typically cause substantial additional fitness decreases through virion production and assembly and eventually host lysis, while plasmids often carry genes that are beneficial to the survival of their hosts under certain circumstances (Dionisio et al., 2005; Jiang et al., 2013). It is thus likely that the CRISPR systems in prokaryotes are more sensitive to viruses than to plasmids. This line of argument is also consistent with our results that the presence of CRISPRs is more enriched in virus-associated than in plasmid-associated genomes.

### The Influence of Associated Viruses, Plasmids, and CRISPR on Genome GC-Content

We then investigated which factors contribute significantly to genome GC-content. Consistent with our previous results (LM analysis, Table 2), we found that genome size was indeed the most significant predictor of GC-content, with a relative importance of almost 99% (LM analysis, Table 3). The presence of plasmids also had a significant influence on GC-content, with a relative importance of 1% (Table 3). The presence/absence of viruses and CRISPR had no significant influence on GC-content by themselves; surprisingly, however, the presence of phages reduced the influence of plasmid presence on GC content.

**TABLE 3:** Relative importance of various factors for GC-content (GC%) in a linear model (LM).

**Dataset**	**Factors**	**Coefficient**	***P-*value**	**Relative importance**
All	Size	4.081	<2 × 10^–16^	99.12%
	Plasmid	–1.423	5.5 × 10^–5^	0.85%
	Virus	–0.089	0.788	0.02%
	CRISPR	0.115	0.656	0.01%
	Virus^∗^plasmid	–0.434	0.438	–
No plasmids	Size	4.132	<2 × 10^–16^	99.85%
	Virus	–0.139	0.678	0.01%
	CRISPR	0.618	0.048	0.14%
No viruses	Size	4.107	<2 × 10^–16^	99.30%
	Plasmid	–1.442	7.7 × 10^–5^	0.60%
	CRISPR	0.528	0.109	0.10%

*The equation of “All” dataset used in the LM is GC% ∼ size + plasmid + virus + CRISPR + virus^∗^plasmid; the equation of “No plasmids” dataset is GC% ∼ size + virus + CRISPR; and the equation of “No viruses” dataset is GC% ∼ size + plasmid + CRISPR.*

We also investigated whether these factors contribute significantly to GC-content when genomes contain no viruses/plasmids. As expected, genome size remained the most significant factor for the prediction of genome GC-content, as shown in Table 3, with a relative importance of around 99%.

As shown in Supplementary Table 3, we find that the number of associated viruses and plasmids contribute significantly to GC-content, but we don’t find clear and consistent trends in GC-content as a function of the number of associated viruses or plasmids (Supplementary Figures 3A–F).

## Discussion

We expected that viruses and plasmids could facilitate genome expansions because they can bring novel DNAs (genes or fragments) into prokaryotic cells that can be integrated into the host genome, while CRISPR immune systems could impair such a process by targeting and eliminating foreign DNAs. However, recent studies presented inconsistent results regarding this topic (Marraffini and Sontheimer, 2008; Makarova et al., 2011; Bikard et al., 2012; Gophna et al., 2015; Watson et al., 2018).

To address this issue, we constructed a comprehensive dataset of prokaryotic genomes and their associations with viruses and plasmids. By dividing genomes into distinct groups according to whether they associated with viruses and/or plasmids and/or contained CRISPRs, we revealed that genomes with viruses or with plasmids were significantly larger than those without, and genome sizes increased with increasing numbers of associated viruses/plasmids. Conversely, virus-associated (but not plasmid-associate) genomes with CRISPRs were significantly smaller in size than those without, regardless of the number of associated viruses. These results confirm that in the long run, viruses and plasmids facilitate genome expansions while CRISPR impairs virus-driven genome expansions. In some cases, prokaryotes could utilize foreign DNAs to expand their metabolic capacities and/or enhance their physiological properties (e.g., antibiotic resistance), leading to genome expansion. Conversely, foreign DNAs that did not have immediate benefits would be unlikely to be incorporated, the genomes tend to stay “small(er).” The “Refusal” process is achieved by defense systems including CRISPR. In addition to the CRISPR systems, there are other known and novel anti-phage defense systems, such as Abi, R-M, toxin/anti-toxin and so on (Doron et al., 2018). There are fitness costs inherent to expanding and limiting the genome size (requires more time and energy), which could have major competitiveness impacts when a given prokaryote is in a highly diverse and competitive environments.

It is worth noting that the CRISPR systems themselves could lead to “genome expansion” through incorporating new spacer sequences into CRISPR arrays. On average a genome can contain ∼40 CRISPR spacers, with total length of ∼1.1 k for all the CRISPR array regions. Despite these modest additions to genome size, we still found that CRISPR-containing genomes were smaller, suggesting that the CRISPR arrays had limited impact on the total genome size.

Genome size evolution has previously been reported to be associated with that of genomic GC-content (Gao et al., 2017). Thus, it appeared possible that virus- and/or plasmid-association has a direct effect not only on genome size but also on GC-content. However, in this study, we found only minor influences of viruses and plasmids on genomic GC-content (Table 3 and Supplementary Table 1). We also split our data into archaea and bacteria, and found similar results in bacteria subgroup not in archaea. This is likely due to the less samples of archaea (Supplementary Tables 4–7).

Our results also imply that CRISPR immune systems might be more sensitive toward invading viruses than plasmids, consistent with the differential fitness burdens brought by the two types of foreign invaders to the hosts (Canchaya et al., 2004; Weinberger et al., 2012; Jiang et al., 2013; Pleska and Guet, 2017).

Our results differ significantly from several previous studies (Gophna et al., 2015; Watson et al., 2018). For example, Gophna et al. (2015) reported that the inhibitory effect of CRISPR against HGT is undetectable using three independent measures of recent HGT. However, it is known that CRISPR spacers – which were used by Gophna et al. (2015) to assess CRISPR activity – have very high turnover rates, on the time-scale of days (Deveau et al., 2008; Horvath et al., 2008; Tyson and Banfield, 2008), while HGT genes may take a very long time to be incorporated into existing gene networks (Lercher and Pal, 2008), suggesting that it is only possible to look at the impacts of CRISPRs on HGTs at evolutionary scales. Interestingly, Gophna et al. (2015) also studied spacer acquisition and concluded there was a bias toward frequently encountered invasive exogenous genetic elements, especially infecting viruses; this is consistent with our conclusion that CRISPRs tend to be more sensitive toward invading viruses than plasmids. Recently, Watson et al. (2018) reported that the CRISPR system of the bacterium *Pectobacterium atrosepticum* enabled the host to resist phage infection, but that this enhanced rather than impeded HGT by transduction. However, it is yet to be seen whether or not this phenomenon is unique to *P. atrosepticum*. Though our findings are known to hold true globally, there will certainly be some exceptions with fewer reports at present.

## Data Availability Statement

The raw data supporting the conclusions of this manuscript will be made available by the authors, without undue reservation, to any qualified researcher.

## Author Contributions

W-HC initialized, conceptualized, and designed the study. NG, JC, and TW analyzed the data, wrote and edited the manuscript. ML contributed to key discussions and methods on findings, and prepared the tables and figures. W-HC and ML edited the manuscript. All authors read and approved the final manuscript.

## Conflict of Interest

The authors declare that the research was conducted in the absence of any commercial or financial relationships that could be construed as a potential conflict of interest.

## Footnotes

1 https://www.ncbi.nlm.nih.gov/genome/; accessed on June 16, 2019.

2 https://crisprcas.i2bc.paris-saclay.fr; last update June 18, 2019.
