# Supplementary figures and images for: Prokaryotic Genome Expansion Is Facilitated by Phages and Plasmids but Impaired by CRISPR
Source: Front Microbiol. 2019 Oct 16;10:2254. doi: 10.3389/fmicb.2019.02254 (PMC6805729; doi:10.3389/fmicb.2019.02254)

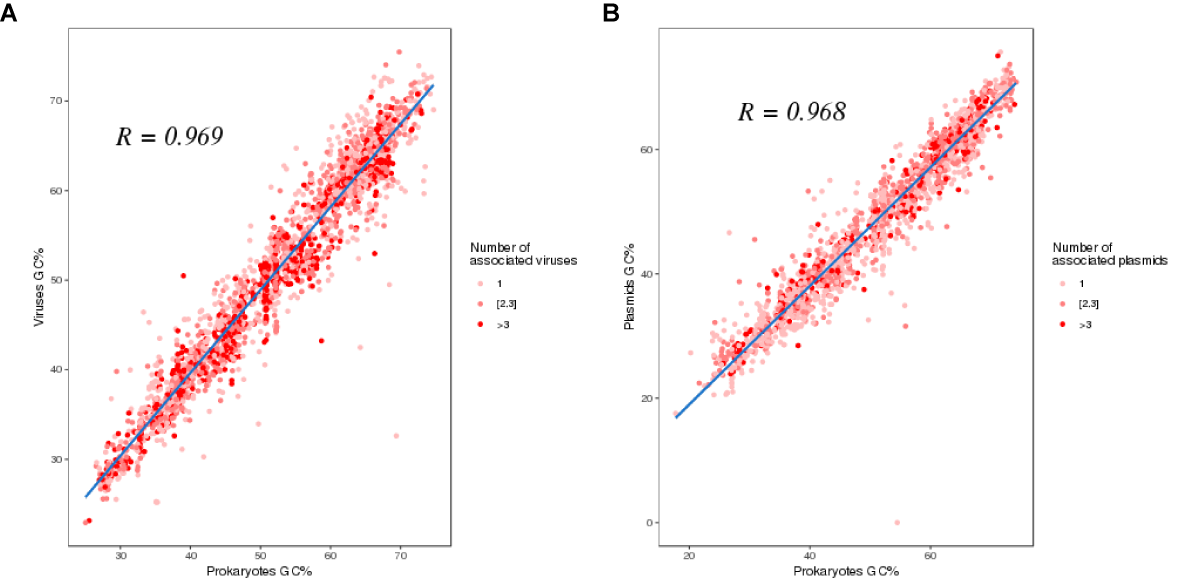

Supplement: Supplementary file 1 [file Image_1.tif]

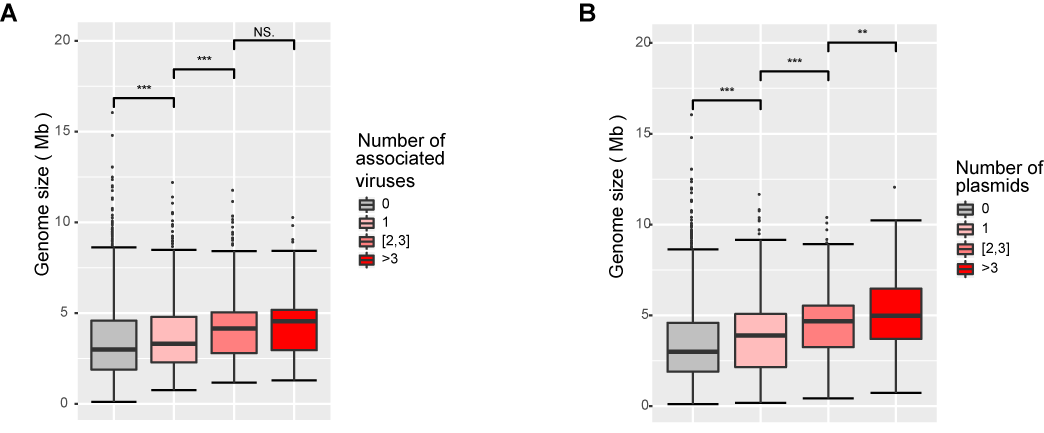

Supplement: Supplementary file 2 [file Image_2.tif]

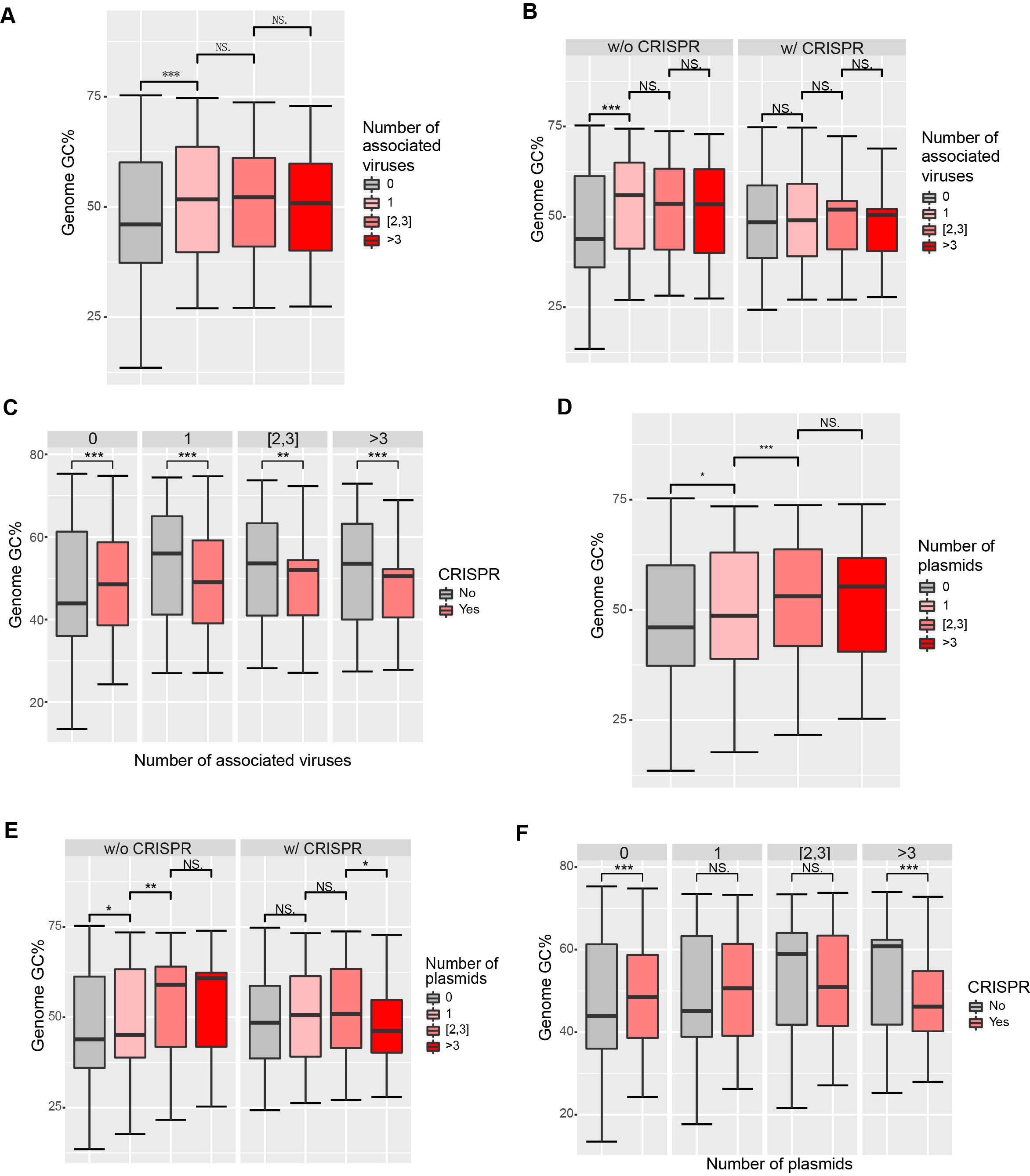

Supplement: Supplementary file 3 [file Image_3.jpg]
