# Supplementary material for: Prokaryotic Genome Expansion Is Facilitated by Phages and Plasmids but Impaired by CRISPR
Source: Front Microbiol. 2019 Oct 16;10:2254. doi: 10.3389/fmicb.2019.02254 (PMC6805729; doi:10.3389/fmicb.2019.02254)
Supplement: Supplementary file 5 [file Table_2.docx]

**Supplementary Figure 1.** Correlation of the GC-contents of the host genomes and their associated viruses (A) and plasmids (B).

**Supplementary Figure 2.** Increasing numbers of viruses and plasmids are associated with increased genome sizes. A) Boxplot of genomes size as a function of the number of associated viruses. Genome sizes are larger with increasing numbers of associated viruses, when genomes encode CRISPR systems. B) Boxplot of genomes size as a function of the number of associated plasmids. The impact of plasmids on genome size is similar to that of viruses.

**Supplementary Figure 3.** No clear trends could be observed in genome GC-content as a function of the number of associated viruses and plasmids. A) Boxplot of genome GC-content as a function of the number of associated viruses. B-C) Boxplots of genome GC-content as a function of the presence/absence of CRISPRs in genomes associated with viruses. The GC-content of virus-associated genomes with CRISPRs are significantly lower than without, regardless of the number of associated viruses. In contrast, in genomes without virus-associations, CRISPR-containing genomes are significantly higher in GC-content than genomes without CRISPRs. D) Boxplot showing the genome GC-content as a function of associated plasmids. E-F) Boxplots of genome GC-content in genomes associated with plasmids as a function of the presence/absence of CRISPRs. Wilcoxon rank sum tests were used to compare between groups. Level of significance: *** *P*<0.001; ** *P*<0.01; * *P*<0.05; NS. *P*≥0.05.

**Supplementary Table 2.** 7,085 prokaryotic genomes and their association with viruses, plasmids and CRISPRs.

|  | **Prokaryotes** | | |
| --- | --- | --- | --- |
|  | **Sum** | **Archaea**  **(304)** | **Bacteria**  **(6781)** |
| **Viruses** | 2682 | 28 (9.2%) | 2654 (39.1%) |
| **Plasmids** | 2221 | 73 (24%) | 2148 (31.7%) |
| **CRISPRs** | 2861 | 224 (73.7%) | 2637 (38.9%) |

**Supplementary Table 3.** Relative importance of various factors for GC-content (GC%) in a LM.

| **Dataset** | **Factors** | **Coefficient** | ***P-*value** | **Relative importance** |
| --- | --- | --- | --- | --- |
| All | size | 4.087 | < 2x10^-16^ | 98.47% |
|  | plasmidNumber | -0.556 | 8.5x10^-11^ | 1.35% |
|  | virusNumber | -0.016 | 0.389 | 0.16% |
|  | CRISPR | -0.209 | 0.412 | 0.02% |
| No plasmids | size | 4.139 | < 2x10^-16^ | 99.76% |
|  | virusNumber | -0.060 | 0.014 | 0.22% |
|  | CRISPR | 0.261 | 0.398 | 0.03% |
| No viruses | size | 4.120 | < 2x10^-16^ | 99.24% |
|  | plasmidNumber | -0.485 | 1.8x10^-5^ | 0.74% |
|  | CRISPR | 0.262 | 0.015 | 0.02% |

Note: The equation used in the linear model (LM) is GC% ~ size + plasmidNumber + virusNumber + CRISPR. Here, plasmidNumber and virusNumber represent the number of plasmids and viruses in genomes, respectively.

**Supplementary Table 4.** Relative importance of various factors for genome size in bacteria.

| **Dataset** | **Factor** | **Coefficient** | ***P-*value** | **Relative importance** |
| --- | --- | --- | --- | --- |
| All | GC% | 0.088 | < 2x10^-16^ | 91.92% |
|  | plasmid | 0.701 | < 2x10^-16^ | 5.84% |
|  | virus | 0.429 | < 2x10^-16^ | 2.24% |
|  | CRISPR | -0.003 | 0.936 | 0.00% |
|  | virus*plasmid | -0.162 | 0.046 | - |
| No plasmids | GC% | 0.088 | < 2x10^-16^ | 96.95% |
|  | virus | 0.432 | < 2x10^-16^ | 3.01% |
|  | CRISPR | -0.052 | 0.263 | 0.05% |
| No viruses | GC% | 0.089 | < 2x10^-16^ | 93.69% |
|  | plasmid | 0.696 | < 2x10^-16^ | 5.96% |
|  | CRISPR | 0.156 | 0.003 | 0.35% |

| **Supplementary Table 5**. Relative importance of various factors for GC-content (GC%) in bacteria. | | | | |
| --- | --- | --- | --- | --- |
| **Dataset** | **Factors** | **Coefficient** | ***P-*value** | **Relative importance** |
| All | size | 4.142 | < 2x10^-16^ | 98.79% |
|  | plasmid | -1.437 | 1.1x10^-4^ | 1.15% |
|  | virus | 0.007 | 0.983 | 0.04% |
|  | CRISPR | -0.258 | 0.326 | 0.02% |
|  | virus*plasmid | -1.023 | 0.067 | - |
| No plasmids | size | 4.179 | < 2x10^-16^ | 99.98% |
|  | virus | -0.047 | 0.887 | 0.00% |
|  | CRISPR | 0.234 | 0.465 | 0.02% |
| No viruses | size | 4.092 | < 2x10^-16^ | 99.41% |
|  | plasmid | -1.484 | 1.5x10^-4^ | 0.59% |
|  | CRISPR | 0.026 | 0.942 | 0.00% |

**Supplementary Table 6.** Relative importance of various factors for genome size in archaea.

| **Dataset** | **Factor** | **Coefficient** | ***P-*value** | **Relative importance** |
| --- | --- | --- | --- | --- |
| All | GC% | 0.017 | 7.88x10^-4^ | 22.10% |
|  | plasmid | 0.790 | 1.17x10^-8^ | 70.97% |
|  | virus | 0.249 | 0.254 | 6.16% |
|  | CRISPR | 0.078 | 0.506 | 0.78% |
|  | virus*plasmid | 0.229 | 0.54 | - |
| No plasmids | GC% | 0.008 | 0.192 | 30.01% |
|  | virus | 0.202 | 0.339 | 16.12% |
|  | CRISPR | 0.236 | 0.081 | 53.87% |
| No viruses | GC% | 0.018 | 7.7x10^-4^ | 26.33% |
|  | plasmid | 0.785 | 4.7x10^-8^ | 71.91% |
|  | CRISPR | 0.110 | 0.382 | 1.74% |

**Supplementary Table 7.** Relative importance of various factors for GC-content (GC%) in archaea.

| **Dataset** | **Factors** | **Coefficient** | ***P-*value** | **Relative importance** |
| --- | --- | --- | --- | --- |
| All | size | 4.056 | 7.88x10^-4^ | 30.27% |
|  | plasmid | 6.394 | < 2x10^-16^ | 56.97% |
|  | virus | -1.930 | 0.876 | 0.73% |
|  | CRISPR | -2.894 | 0.535 | 12.03% |
|  | virus*plasmid | 8.953 | 0.036 | - |
| No plasmids | size | 0.996 | 0.192 | 67.82% |
|  | virus | -2.184 | 0.369 | 32.18% |
|  | CRISPR | 0.004 | 0.998 | 0.00% |
| No viruses | size | 2.266 | 7.7x10^-4^ | 37.26% |
|  | plasmid | 6.362 | 9.9x10^-5^ | 50.31% |
|  | CRISPR | -2.741 | 0.05 | 12.44% |
